# Impairment of humoral and cellular immune response to tetanus toxoid in HTLV-1 infected individuals

**DOI:** 10.1186/1742-4690-11-S1-P66

**Published:** 2014-01-07

**Authors:** Anselmo S Souza, Camila F Amorim, Natália Carvalho, Silvane B Santos, Edgar M Carvalho

**Affiliations:** 1 Immunology Service, Federal University of Bahia, Salvador, Bahia, BR, Brazil; 2 Natiotal Institute of Science and Technology - Tropical Diseases, Salvador, Bahia, BR, Brazil; 3 Institute of Biological Science, State University of Feira de Santana, Feira de Santana, Bahia, BR, Brazil

## 

The human T cell lymphotropic virus type-1 (HTLV-1) infect mainly T cells, dendritic cells and macrophages inducing T cell proliferation and increasing production of chemokines and cytokines by peripheral blood mononuclear cells (PBMC). However, as HTVL-1 may modify the immune response to other infectious agents, the aim of this study was to evaluate the humoral and cellular immune response before and after vaccination with tetanus toxoid (TT). Participants included 14 HTLV-1 carriers and 12 healthy subjects (HS). These individuals were immunized with two doses of tetanus toxoid vaccine. Antibodies to TT were determined by ELISA and the frequency of T cells expressing cytokines as well as the frequency of monocytes expressing co-stimulatory molecules were determined by FACS. The IgG titers anti-TT increased after immunization in both groups (p = 0.001), but HTLV-1 patients had lower levels of IgG anti-TT after immunization when compared with HS (p = 0.007). The frequency of CD4+ T cells expressing IFN-g, TNF and IL-10, after stimulation with TT, was lower in HTLV-1 infected subjects than the HS after immunization (p < 0.05). TNF and IL-12 expression by monocytes after stimulation with TT were higher in the HTLV-1 group than in HS. However there was an impairment in the HLA-DR expression by monocytes from HTLV-1 infected subjects. These results indicate that HTLV-1 infected subjects have a decreasing in humoral response and an impairment in both antigen presenting cells and T cell functions to a biased antigen. Financial Support: INCT-DT, CNPq, NIH R01.

